# An Introductory Tutorial on Propensity Score Matching in RStudio Using the National Health Interview Survey

**DOI:** 10.7759/cureus.112374

**Published:** 2026-07-09

**Authors:** Rakchhya Uprety, Faith Ogini, La'Marcus T Wingate

**Affiliations:** 1 Department of Pharmaceutical Sciences, Howard University, Washington DC, USA; 2 Department of Clinical and Administrative Pharmacy Sciences, Howard University, Washington DC, USA

**Keywords:** nhis dataset, propensity score matching, rstudio software, telehealth, tutorial

## Abstract

Propensity score matching (PSM) is a statistical method that is used to reduce bias due to confounding from observational studies or studies where randomization is not possible by matching the treatment groups to the control groups based on their propensity scores. A propensity score is a value that gives the probability of each individual subject being assigned to a specific treatment group based on the covariates selected. The aim of this paper is to provide a step-by-step tutorial that demonstrates how to create a propensity-matched cohort using RStudio (Posit PBC, Boston, MA). For this tutorial, we utilize the publicly available 2022 National Health Interview Survey adult dataset. Our focus is on individuals residing in non-metropolitan areas. In our study, the propensity scores represent the probability that a patient uses telehealth, and it is estimated using multiple logistic regression, where telehealth usage is the dependent variable and the predictor variables are age, sex, race, income-to-poverty ratio, education, marital status, insurance status, communication difficulty, and Hispanic ethnicity. This analysis demonstrates how PSM can improve balance in measured covariates and may help reduce bias from observed confounders. Several covariates in the analysis had standardized mean differences (SMDs) greater than 0.1 before matching. However, after PSM, all the covariates achieved SMDs below 0.1, which indicates a better balance at baseline between groups. As this is a pedagogical tutorial, results should not be interpreted as a nationally representative matched analysis.

## Introduction

Confounding is a type of bias that occurs when the relationship between an independent variable (exposure) and the dependent variable (outcome) is influenced by a confounding variable [1]. For a variable to be considered a confounder, it should be associated with exposure as well as the outcome variable, and it should not be an intermediate in the causal pathway [2]. The presence of a confounder can make it appear that there is an association between the treatment and outcome when no real association exists between them [2].

In randomized controlled trials (RCTs), confounding is minimized through randomization that leads to a balance in the baseline covariates between treatment and control groups [3]. However, in observational studies, there is no randomization, which can lead to uneven distribution of treatment and control groups [4]. While propensity score matching (PSM) remains one of the most commonly used approaches for confounding adjustment, other propensity score (PS) methods are available, including inverse probability of treatment weighting (IPTW), PS stratification, and PS adjustment [5]. Furthermore, newer causal inference approaches such as targeted maximum likelihood estimation (TMLE) and g-computation have also been increasingly applied in observational research. It is also important to note that there is an ongoing debate about the use of PSM in observational studies, as some researchers argue that PSM has its limitations, including potential loss in sample size, increased statistical bias, inability to address unmeasured confounding, and model dependence.

Good control of confounding is supported when baseline characteristics are well balanced between comparison groups. For example, in an RCT, a table showing similar general or baseline characteristics between groups helps demonstrate that randomization was successful in minimizing confounding [6]. Similarly, in observational studies, a well-balanced table that includes standardized mean differences (SMDs) less than 0.1 across covariates can provide evidence for good control of confounding [7].

PS can be used to minimize the effects of confounding and to help ensure that the groups are similar to each other regarding baseline characteristics. A PS represents the probability that an individual would receive a treatment based on their baseline characteristics [8]. The PS value ranges from 0 to 1, and these scores can be calculated using logistic regression in which the outcome variable is the treatment assignment and the predicted variables are the observed baseline characteristics [8]. These scores are used to match the treatment group with the controls. PSM pairs each subject in the intervention group with one or more subjects in the comparison group (e.g., 1:1 or 1:k matching) based on similarity in propensity scores [9].

One-to-one or one-to-two matching is the most commonly used method of PSM, where each treated individual is matched with one or two control individuals who have similar propensity scores. PSM can be done with or without replacement. Matching with replacement allows the same control to be included in more than one matched set [8]. When each control is included in only one matched set and cannot be used again, it is matching without replacement, and it is more frequently used than with replacement [9].

Nearest neighbor matching is a commonly used method for selecting the matches based on the scores, and as the name suggests, individuals can be paired with one or more controls whose propensity scores are closest in value [8]. Another important concept in PSM is the use of a caliper, which refers to the maximum allowable difference in propensity scores between matched individuals [10]. In nearest neighbor matching with a caliper, a treated subject is only matched to a control subject if the difference in their propensity scores is within a specified range (caliper), typically expressed as a fraction (e.g., 0.2) of the standard deviation of the logit of the propensity score [11]. This restriction helps to improve the quality of the matches by avoiding poor matches with substantially different scores, reducing bias. However, using a very narrow caliper may lead to fewer matches and a reduced sample size, thus representing a tradeoff between power and similarity between the treatment and control groups [9]. To evaluate how well the treatment and comparison groups are balanced after matching, researchers often examine the standardized differences in means or proportions of baseline characteristics. While there is no universally accepted cutoff, a commonly used guideline is that a standardized difference of less than 0.1 indicates good balance between the groups [12]. Researchers can further test whether the matching achieved its intended purpose by comparing the distribution of propensity scores between the two groups before and after matching.

## Technical report

Accessing the software and the dataset

For this tutorial, we used RStudio (Posit PBC, Boston, MA), a free and open-source statistical software widely used for data analysis. The version we have used is 4.1.1. It can be downloaded from https://posit.co/download/rstudio-desktop/ and installed on the user’s device [13]. In order to follow this tutorial, we assume that you have basic RStudio knowledge. The various packages used for this demonstration are explained in Table 1 below.

**Table 1:** List of RStudio packages used for demonstration of propensity score matching in a national dataset.

R Package	Description
MatchIt	Implements propensity score matching methods.
Ggplot2	Creates data visualization and graphics.
Dplyr	Used for data manipulation with functions like filter, select, and mutate.
Tableone	Creates ‘Table 1’ summary tables to describe baseline characteristics in medical research.
Readr	Provides fast and friendly functions for reading rectangular data like CSV files.
Haven	Allows loading data from other statistical packages like SAS, SPSS, and STATA.
Gtsummary	Help create publication-ready analytical and summary tables.

The dataset in this tutorial is the 2022 National Health Interview Survey (NHIS) adult dataset, and it is freely available from https://www.cdc.gov/nchs/nhis/documentation/2022-nhis.html [14]. Open this link, scroll down to public use data under sample adult interview, and download the CSV data file. This dataset is a nationally representative survey that provides information on disease conditions, insurance coverage, healthcare access, disabilities, etc., of the non-institutionalized US population. In this demonstration, we use the dataset to create a matched sample based on telehealth use. The outcome variable is the use of telehealth, and the population is the subgroup of individuals who live in non-metropolitan areas. The variable for use of telehealth in the past 12 months in the dataset is VIRAPP12M_A, where 1 corresponds to "Yes", and 2 represents "No". The values 7, 8, and 9 correspond to refused, not ascertained, and don’t know, respectively. The geographic location is classified as either metropolitan or non-metropolitan by the variable URBRRL (Urban-Rural Classification Variable in NHIS). The other variables we are including in this analysis to adjust for confounders are age (AGEP_A), sex (SEX_A), race (RACEALLP_A), education (EDUCP_A), marital status (MARITAL_A), insurance coverage (HICOV_A), communication difficulty (COMDIFF_A), poverty status (POVRATTC_A), and ethnicity (HISP_A).

**Figure 1:**
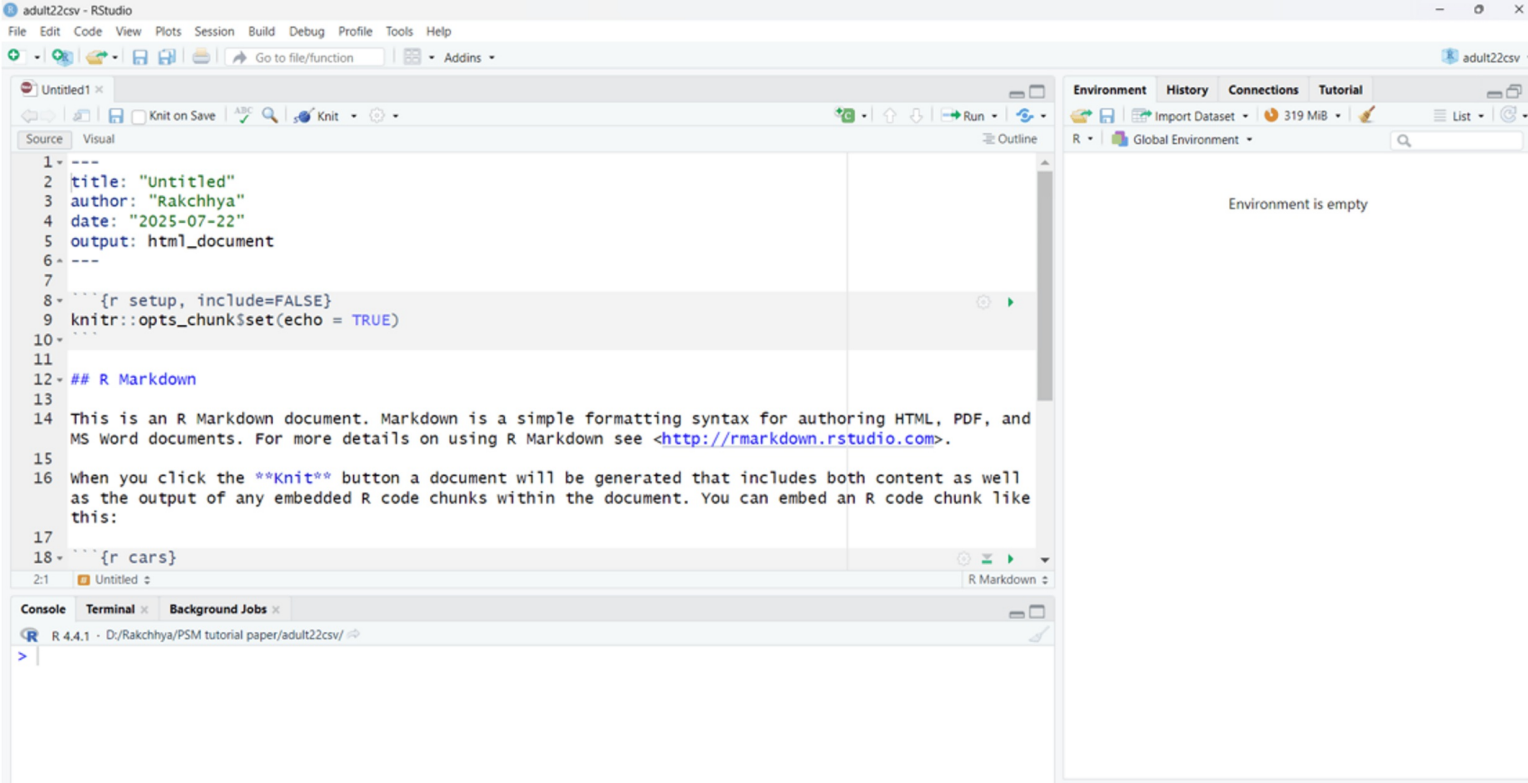
Screenshot of the RStudio interface with an open R Markdown file used for this tutorial.

After your RStudio has been installed, to create a new *R Markdown* document, click on *File* in the top left corner, as seen in Figure 1 below. Then click on *New File*, followed by *R Markdown*. In the dialogue box, you can proceed to provide a title and author name.

**Figure 2:**
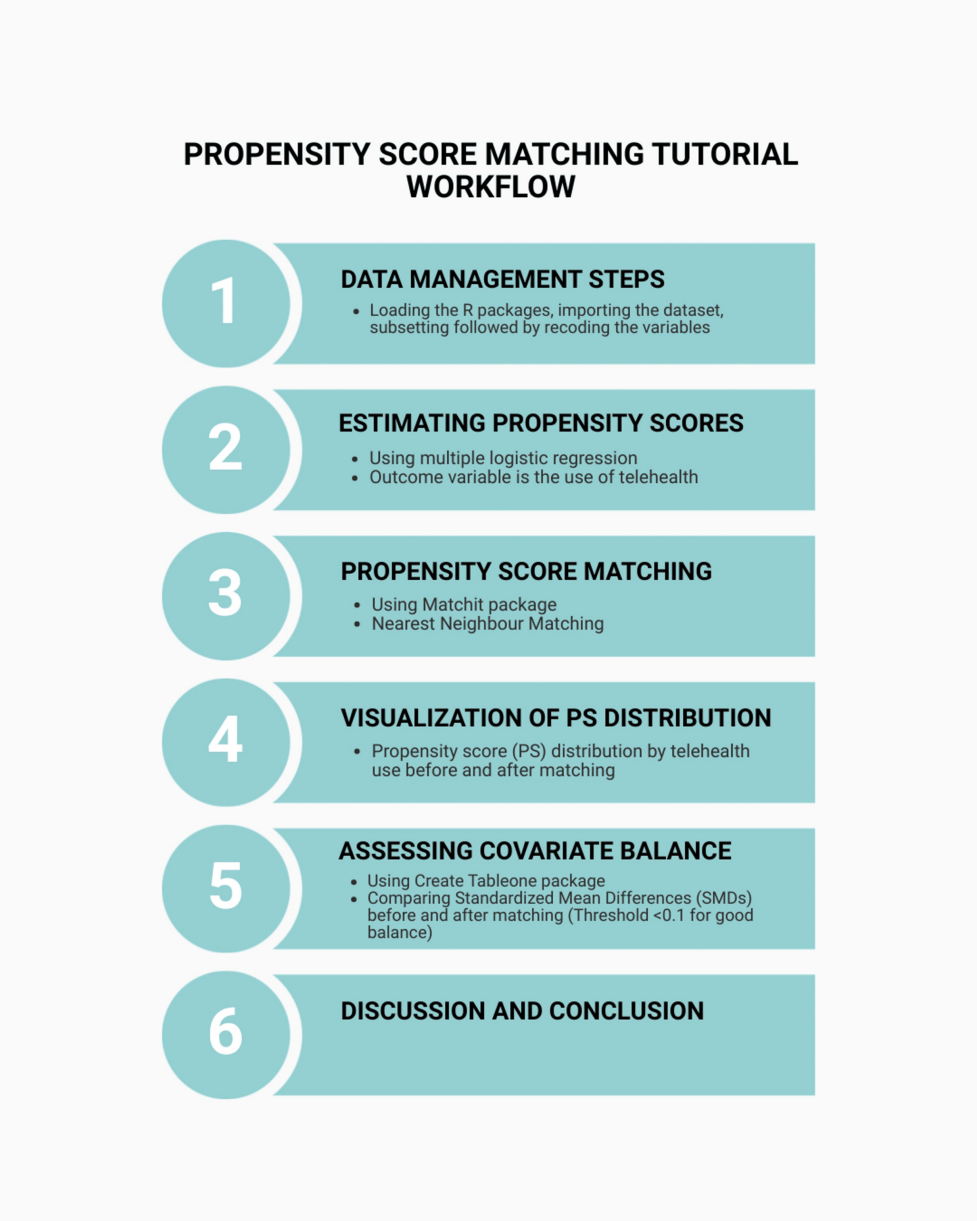
Propensity score matching tutorial: Workflow diagram.

The next step would be to install the required packages using the function *install.packages* if you have not installed them previously. For the next step, you can load all the indicated packages with the library command. You can simply copy and paste the codes below and run them. In order to run them, you need to click on the small green run button in the right corner of that chunk. The steps this tutorial follows are explained in Figure 2 below as a workflow diagram.

install.packages("MatchIt") # For propensity score matching install.packages("ggplot2") # For data visualization install.packages("dplyr") # For data manipulation install.packages("tableone") # For creating summary tables install.packages("readr") # For reading data install.packages("haven") # For loading dataset install.packages("gtsummary") # For creating summary tables 

library(MatchIt) library(ggplot2) library(dplyr) library(tableone) library(haven) library(gtsummary) library(readr) 

Loading the Dataset Into RStudio

The next step would be to load the data into RStudio. For this, we use the downloaded CSV file from the link above. Click on the link above, scroll down to “sample adult interview”, then click on “public use data” and download the CSV (adult22.csv) data file. We will use *readr* to load the dataset. This dataset will now be renamed as TelehealthNHIS. You have to replace the text that says “path_to_your_file” in the code below with the actual path where the file is saved on your computer.

TelehealthNHIS<-read_csv("path/to/your/file/adult22.csv") View(TelehealthNHIS) 

Data management steps

Subsetting and Recoding the Variables

The code below is used to manage the data and ensure that the subset is created appropriately. In this demonstration, the subset we are interested in is individuals who live in non-metropolitan (rural) areas, and according to the NHIS codebook 2022, a non-metropolitan area corresponds to URBRRL = 4. We have also recoded the variable VIRAPP12M_A and created a new variable called telehealth_use, where 1 = used telehealth and 0 = did not use telehealth in the past 12 months.

OnlyRural<-TelehealthNHIS%>% filter(URBRRL==4) #To check filtering is appropriate table(TelehealthNHIS$URBRRL) table(OnlyRural$URBRRL) glimpse(OnlyRural) #We have two groups: Those who used telehealth in the past 12 months (VIRAPP12M_A=1) and those who did not (VIRAPP12M_A=2) telehealth_counts <- table(OnlyRural$VIRAPP12M_A) # Display the counts print(telehealth_counts) # 7,8 and 9 are missing as per the codebook OnlyRural <- OnlyRural %>% mutate(telehealth_use = ifelse(VIRAPP12M_A == 1, 1, ifelse(VIRAPP12M_A == 2, 0, NA))) #removes 7,8 and 9 telehealth_use <- table(OnlyRural$telehealth_use)

# Display the counts print(telehealth_use) #outcome variable

Furthermore, we need to wrangle the dataset and recode the variables to make it easier to interpret the findings. For example, race variables are recoded as White, Black, and other races. Education is reclassified as less than a bachelor’s degree and as a bachelor’s degree or higher. Marital status is recoded as married or unmarried.

OnlyRural <- OnlyRural %>% mutate( SEX_AR = ifelse(SEX_A == 1, 1, #Male ifelse(SEX_A == 2, 0, NA)), #Female # Remove missing RACEALLP_AR = ifelse(RACEALLP_A == 1, 1, # White ifelse(RACEALLP_A == 2, 2, # Black ifelse(RACEALLP_A %in% c(3,4,5,6), 3, NA))), # All other races EDUCP_AR = ifelse(EDUCP_A %in% c(8, 9, 10), 1, # Bachelor's degree or more ifelse(EDUCP_A %in% 1:7, 0, NA)), # Less than bachelor's MARITAL_AR = ifelse(MARITAL_A == 1, 1, # Married ifelse(MARITAL_A %in% c(2, 3), 0, NA)), # Unmarried HICOV_AR = ifelse(HICOV_A == 1, 1, # Insured ifelse(HICOV_A == 2, 0, NA)), # Not insured COMDIFF_AR = ifelse(COMDIFF_A == 1, 0, # No difficulty ifelse(COMDIFF_A %in% c(2, 3, 4), 1, NA)), HISP_AR = ifelse(HISP_A == 1, 1, # Hispanic ifelse(HISP_A == 2, 0, NA)) # Non-Hispanic) 

Filtering the Dataset to Include Only Variables of Interest

Since the dataset has an extensive number of variables, we select only the variables of interest to make the analysis easier. The missing values are handled using the complete case analysis (CCA) method, wherein cases with missing values are removed, as in our dataset [15]. The CCA was used here for its pedagogical simplicity, allowing readers to follow the steps of propensity score estimation. Before filtering the dataset for missing values, we had 4311 observations, and after removing observations with missing data, the new dataset (mydata_clean) has 4085 observations.

mydata_onlyRural <- OnlyRural %>%

 select(telehealth_use,AGEP_A,SEX_AR,RACEALLP_AR,POVRATTC_A,EDUCP_AR,MARITAL_AR,HICOV_AR,COMDIFF_AR,HISP_AR)

#To see if the variables are numeric and character and if there are any missing data

glimpse(mydata_onlyRural)

#Filter outcome variable to exclude missing

mydata_clean <- mydata_onlyRural %>%

filter(if_all(c(telehealth_use,AGEP_A,SEX_AR,RACEALLP_AR,POVRATTC_A,EDUCP_AR,MARITAL_AR,HICOV_AR,COMDIFF_AR,HISP_AR),~!is.na(.)))

glimpse(mydata_clean)

We also need to convert the categorical variables to factors to ensure that R treats them as categorical variables and not as continuous variables, which could lead to incorrect estimation of propensity scores.

mydata_clean <- mydata_clean %>%

 mutate(

 telehealth_use = factor(telehealth_use, levels = c(0, 1), labels = c("No", "Yes")),

 SEX_AR = factor(SEX_AR, levels = c(0, 1), labels = c("Female", "Male")),

 RACEALLP_AR = factor(RACEALLP_AR, levels = c(1, 2, 3), labels = c("White", "Black", "Other")),

 EDUCP_AR = factor(EDUCP_AR, levels = c(0, 1), labels = c("Less than Bachelor's", "Bachelor's or more")),

 MARITAL_AR = factor(MARITAL_AR, levels = c(0, 1), labels = c("Unmarried", "Married")),

 HICOV_AR = factor(HICOV_AR, levels = c(0, 1), labels = c("Not Insured", "Insured")),

 COMDIFF_AR = factor(COMDIFF_AR, levels = c(0, 1), labels = c("No Difficulty", "Difficulty")),

 HISP_AR = factor(HISP_AR, levels = c(0, 1), labels = c("Non-Hispanic", "Hispanic")))

glimpse(mydata_clean)

Creating propensity scores

In order to fit a propensity score model, we use logistic regression, where the use of telehealth is a binary outcome variable. Here, those who used telehealth are the experimental group, and those who did not are the control group. The variable “telehealth_use” represents the treatment assignment and is not included as a covariate in the matching process itself but is used as a dependent variable in the propensity score model to estimate each individual’s probability of receiving the treatment based on the covariates selected. Family binomial in the code below denotes a binary outcome. The estimate will inform us who is more likely to use telehealth based on the baseline characteristics

#telehealth_use is the treatment indicator

psmodel <- glm(telehealth_use ~ AGEP_A + SEX_AR + RACEALLP_AR + POVRATTC_A +

 EDUCP_AR + MARITAL_AR + HICOV_AR + COMDIFF_AR + HISP_AR,

 family = binomial(), data = mydata_clean)

summary(psmodel)

pscore <- psmodel$fitted.values

# Add propensity scores to the dataset

mydata_clean_pscore <- mydata_clean %>%

 mutate(pscore = pscore)

Now, if you look at the new dataset mydata_clean_pscore, you will notice that you will have 11 variables, and the last column, p score, has propensity scores for each observation, which range from 0 to 1.

Visualizing the Distribution of Propensity Scores Before Matching

The PS distribution can also be visualized using a histogram. To see how PSM affects our data, let us visualize the distribution of propensity scores before matching using ggplot and plot a histogram.

ggplot(mydata_clean_pscore, aes(x = pscore, fill = factor(telehealth_use))) +

 geom_histogram(binwidth = 0.05, alpha = 0.5, color = "black") +

 labs(title = "Distribution of Propensity Scores Before Matching",

 x = "Propensity Score",

 y = "Count",

 fill = "Telehealth Use")

**Figure 3:**
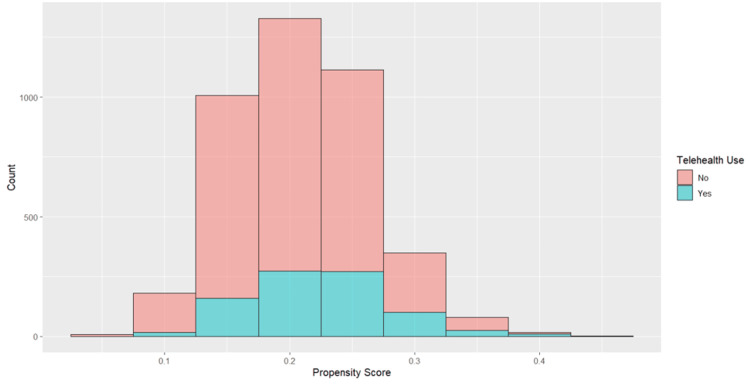
Distribution of propensity scores by telehealth use before propensity score matching.

Figure 3 below displays the distribution of propensity scores before matching for those who used telehealth (green) vs. those who did not use telehealth (pink).

Propensity Score Matching

The *Matchit* package in RStudio allows us to match similar respondents. We utilize the nearest neighbor method to match individuals within the treatment and control groups based on propensity scores. Nearest neighbor matching is known as greedy matching, and it provides a way to create a matched sample by pairing treated and control individuals with the closest propensity scores. We used this method due to its conceptual simplicity and its frequent use in applied health research. There are several other matching methods for PSM, like nearest neighbor matching, optimal pair matching, full matching, exact matching, etc., and choosing the right method depends on the type of data as well as the goal of the analysis [16]. If the researcher wants to use a different matching method, they will have to change method = "nearest" to the matching method of their choice, e.g., method = "optimal". Hence, in this demonstration, the method is set to "nearest", although this command can be modified based on the type of matching the analyst wishes to employ. The code below performs a 1:1 matching, which is the default, which means that each treated individual (in this case, those who used telehealth) will be matched to one control (those who did not use telehealth) with a similar propensity score.

#1:1 nearest neighbor matching without replacement

mydata_matched <- matchit(telehealth_use ~ AGEP_A + SEX_AR + RACEALLP_AR + POVRATTC_A + EDUCP_AR + MARITAL_AR + HICOV_AR + COMDIFF_AR + HISP_AR,

 data=mydata_clean_pscore,

 method="nearest")

matched_data_output <- match.data(mydata_matched)

Visualizing the Distribution of Propensity Scores After Matching

In order to visualize the distribution of propensity scores after matching, we used the matched dataset (matched_data_output) and the code below.

ggplot(matched_data_output, aes(x = pscore, fill = factor(telehealth_use))) +

 geom_histogram(binwidth = 0.05, alpha = 0.5, color = "black") +

 labs(title = "Distribution of Propensity Scores After Matching",

 x = "Propensity Score",

 y = "Count",

 fill = "Telehealth Use")

**Figure 4:**
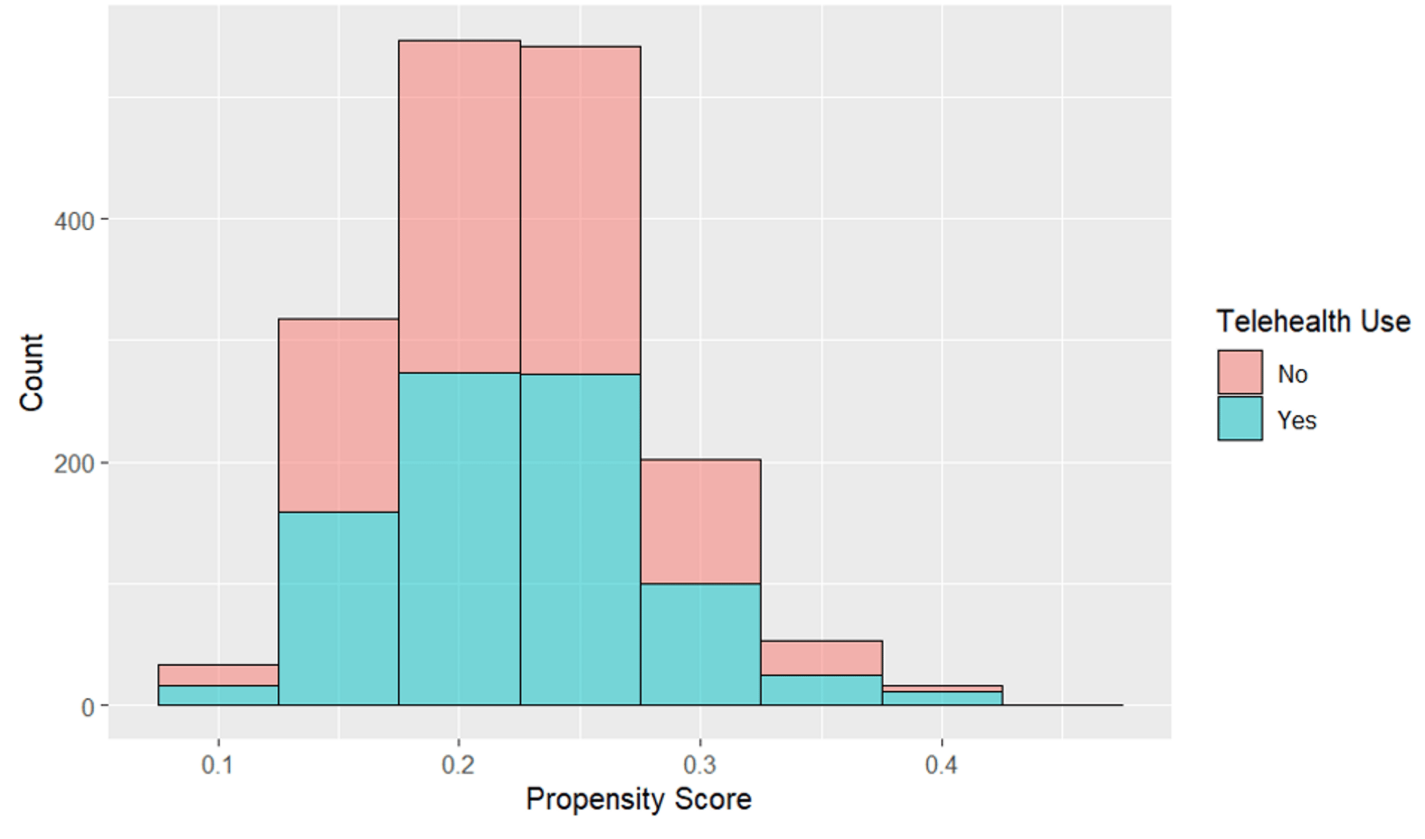
Distribution of propensity scores by telehealth use after propensity score matching.

After matching, the propensity scores for both groups are more similar and have a better overlap with similar shapes and ranges, as shown in Figure 4, compared to the distribution shown in Figure 3.

Covariate Balance Assessment After Propensity Score Matching

After creating a matched dataset, it is particularly important to check if the covariates are balanced at baseline. This can be done by comparing the SMDs between groups, i.e., treatments and control. In general, when the SMD is less than 0.1, the groups are considered to be well balanced at baseline [7]. We can create a summary table stratified by telehealth use via the Tableone package. Within our programming statements, we use the telehealth variable within the strata = command to stratify by telehealth. We can now see how the distribution of the variables and the SMD compare between the treatment and control groups.

# Selecting variables for summary table

xvars <- c("AGEP_A", "SEX_AR", "RACEALLP_AR", "POVRATTC_A",

 "EDUCP_AR", "MARITAL_AR", "HICOV_AR", "COMDIFF_AR",

 "HISP_AR")

# Create summary table for unmatched data

unmatchedtab <- CreateTableOne(vars = xvars, strata = "telehealth_use", data = mydata_clean_pscore, test = FALSE)

# Print the table with standardized mean differences (SMD)

print(unmatchedtab, smd = TRUE,showAllLevels=TRUE)

From Table 2, we can see that the SMD for the variables sex, race, insurance coverage, and difficulty in communication is greater than the threshold of 0.1. Hence, it can be said that these four variables are not well balanced at baseline.

**Table 2:** Summary table of the variables stratified by telehealth use, along with the standardized mean differences (SMD) for the unmatched dataset.

Variable	Level	No (n = 3228)	Yes (n = 857)	SMD
AGEP_A (mean (SD))		56.22 (18.33)	56.24 (16.93)	0.001
SEX_AR (%)	Female	1664 (51.5)	527 (61.5)	0.202
	Male	1564 (48.5)	330 (38.5)	
RACEALLP_AR (%)	White	2810 (87.1)	753 (87.9)	0.105
	Black	261 (8.1)	50 (5.8)	
	Other	157 (4.9)	54 (6.3)	
POVRATTC_A (mean (SD))		3.23 (2.38)	3.15 (2.37)	0.033
EDUCP_AR (%)	Less than a bachelor's	2546 (78.9)	641 (74.8)	0.097
	Bachelor's or more	682 (21.1)	216 (25.2)	
MARITAL_AR (%)	Unmarried	1700 (52.7)	424 (49.5)	0.064
	Married	1528 (47.3)	433 (50.5)	
HICOV_AR (%)	Not insured	243 (7.5)	37 (4.3)	0.136
	Insured	2985 (92.5)	820 (95.7)	
COMDIFF_AR (%)	No difficulty	3012 (93.3)	773 (90.2)	0.113
	Difficulty	216 (6.7)	84 (9.8)	
HISP_AR (%)	Non-Hispanic	3123 (96.7)	819 (95.6)	0.061
	Hispanic	105 (3.3)	38 (4.4)	
Hispanic	105 (3.3)	38 (4.4)		

The next step would be to create a summary table for the matched dataset and compare the SMD across the groups.

# Summary table for matched data

matchedtab1 <- CreateTableOne(vars = xvars, strata = "telehealth_use", data = matched_data_output, test = FALSE)

# Print the table with standardized mean differences (SMD)

print(matchedtab1, smd = TRUE, showAllLevels = TRUE)

From the results in Table 3, we can see that all the variables are well balanced at baseline after matching, as the SMD for all of them is less than 0.1. When you compare Table 2 and Table 3 directly, the largest improvements in balance occurred for sex (SMD reduced from 0.202 to 0.019), insurance coverage (0.136 to 0.012), and race (0.105 to 0.049), consistent with the intended purpose of PSM. One thing to note would be that the sample size of those who did not use telehealth is now 857 from 3228 (Table 2). This is because we conducted a 1:1 matching. If we have enough sample size for controls, we can do even one-to-many matching.

**Table 3:** Summary table of the variables stratified by telehealth use along with the standardized mean differences (SMD) for the 1:1 matched dataset.

Variable	Level		No (n = 857)	Yes (n = 857)	SMD
AGEP_A (mean (SD))			56.25 (18.82)	56.24 (16.93)	0.001
SEX_AR (%)	Female		519 (60.6)	527 (61.5)	0.019
	Male		338 (39.4)	330(38.5)	
RACEALLP_AR (%)	White		741 (86.5)	753 (87.9)	0.049
	Black		60 (7.0)	50 (5.8)	
	Other		56 (6.5)	54 (6.3)	
POVRATTC_A (mean (SD))			3.09 (2.27)	3.15 (2.37)	0.024
EDUCP_AR (%)	Less than a bachelor's		651 (76.0)	641 (74.8)	0.027
	Bachelor's or more		206 (24.0)	216 (25.2)	
MARITAL_AR (%)	Unmarried		421 (49.1)	424 (49.5)	0.007
	Married		436 (50.9)	433 (50.5)	
HICOV_AR (%)	Not insured		35 (4.1)	37 (4.3)	0.012
	Insured		822 (95.9)	820 (95.7)	
COMDIFF_AR (%)	No difficulty		765 (89.3)	773 (90.2)	0.031
	Difficulty		92 (10.7)	84 (9.8)	
HISP_AR (%)	Non-Hispanic		817 (95.3)	819 (95.6)	0.011
	Hispanic		40 (4.7)	38 (4.4)	

If you want to perform 1:2 matching, which means that each treated individual is matched with two controls based on their propensity scores, you can use the code below.

#1:2 nearest neighbor matching without replacement

mydata_matched2 <- matchit(telehealth_use ~ AGEP_A + SEX_AR + RACEALLP_AR + POVRATTC_A + EDUCP_AR + MARITAL_AR + HICOV_AR + COMDIFF_AR + HISP_AR,

 data=mydata_clean_pscore,

 method="nearest", ratio=2) #performs 1:2 matching

matched_data_output2 <- match.data(mydata_matched2)

If you want to perform 1:3 matching, the ratio will have to be changed to 3. Now, we want to create a summary table for a 1:2 matched dataset and see what changed. From Table 4, we can see that the sample size for the treatment (Telehealth “Yes”) group is 857 and that of the control (Telehealth “No”) is 1714, which is double that of the treatment group, which means that 1:2 matching was done. If you compare that to Table 5, where both groups are 857, indicating 1:1 matching.

**Table 4:** Summary table of the variables stratified by telehealth use along with the standardized mean differences (SMD) for the 1:2 matched dataset.

Variable	Level	No (n = 1714)	Yes (n = 857)	SMD
AGEP_A (mean (SD))		56.36 (18.49)	56.24 (16.93)	0.007
SEX_AR (%)	Female	1058 (61.7)	527 (61.5)	0.005
	Male	656 (38.3)	330 (38.5)	
RACEALLP_AR (%)	White	1498 (87.4)	753 (87.9)	0.02
	Black	108 (6.3)	50 (5.8)	
	Other	108 (6.3)	54 (6.3)	
POVRATTC_A (mean (SD))		3.22 (2.37)	3.15 (2.37)	0.03
EDUCP_AR (%)	Less than a bachelor's	1281 (74.7)	641 (74.8)	0.001
	Bachelor's or more	433 (25.3)	216 (25.2)	
MARITAL_AR (%)	Unmarried	824 (48.1)	424 (49.5)	0.028
	Married	890 (51.9)	433 (50.5)	
HICOV_AR (%)	Not insured	75 (4.4)	37 (4.3)	0.003
	Insured	1639 (95.6)	820 (95.7)	
COMDIFF_AR (%)	No difficulty	1545 (90.1)	773 (90.2)	0.002
	Difficulty	169 (9.9)	84 (9.8)	
HISP_AR (%)	Non-Hispanic	1645 (96.0)	819 (95.6)	0.02
	Hispanic	69 (4.0)	38 (4.4)	

**Table 5:** Summary table of the variables stratified by telehealth use along with the standardized mean differences (SMD) for the 1:1 matching with caliper.

Variable	Level	No (n = 853)	Yes (n = 853)	SMD
AGEP_A (mean (SD))		56.24 (18.81)	56.29 (16.93)	0.003
SEX_AR (%)	Female	515 (60.4)	523 (61.3)	0.019
	Male	338 (39.6)	330 (38.7)	
RACEALLP_AR (%)	White	739 (86.6)	751 (88.0)	0.049
	Black	60 (7.0)	50 (5.9)	
	Other	54 (6.3)	52 (6.1)	
POVRATTC_A (mean (SD))		3.10 (2.27)	3.15 (2.38)	0.021
EDUCP_AR (%)	Less than a bachelor's	649 (76.1)	639 (74.9)	0.027
	Bachelor's or more	204 (23.9)	214 (25.1)	
MARITAL_AR (%)	Unmarried	418 (49.0)	424 (49.7)	0.014
	Married	435 (51.0)	429 (50.3)	
HICOV_AR (%)	Not insured	35 (4.1)	37 (4.3)	0.012
	Insured	818 (95.9)	816 (95.7)	
COMDIFF_AR (%)	No difficulty	763 (89.4)	772 (90.5)	0.035
	Difficulty	90 (10.6)	81 (9.5)	
HISP_AR (%)	Non-Hispanic	815 (95.5)	816 (95.7)	0.006
	Hispanic	38 (4.5)	37 (4.3)

# Create summary table for matched data

matchedtab2 <- CreateTableOne(vars = xvars, strata = "telehealth_use", data = matched_data_output2, test = FALSE)

# Print the table with standardized mean differences (SMD)

print(matchedtab2, smd = TRUE, showAllLevels = TRUE)

Propensity Score Matching with a Caliper

One of the major limitations of nearest neighbor matching is that it can give bad matches if the nearest neighbor is far away. To prevent this from happening, we can set a caliper, which represents the maximum allowable difference between the propensity scores of matched observations [10]. A commonly recommended caliper width is 0.2 times the standard deviation of the logit of the propensity score [11]. In this analysis, we will now demonstrate how to do PSM with a caliper. We are using the same PSM method as before, but the key difference is that we set a caliper of 0.2 with the caliper = 0.2 statement incorporated in our code.

# Matching with a caliper of 0.2

match_with_caliper <- matchit(telehealth_use ~ AGEP_A + SEX_AR + RACEALLP_AR + POVRATTC_A + EDUCP_AR + MARITAL_AR + HICOV_AR + COMDIFF_AR + HISP_AR,

 data = mydata_clean_pscore,

 method = "nearest",

 caliper = 0.2)

# Summary of matching results

summary(match_with_caliper)

matched_with_caliper <- match.data(match_with_caliper)

# Create summary table for matched data with caliper

matchedtab2 <- CreateTableOne(vars = xvars, strata = "telehealth_use", data = matched_with_caliper, test = FALSE)

# Print the table with standardized mean differences (SMD)

print(matchedtab2, smd = TRUE, showAllLevels = TRUE)

#propensity score plots

plot(match_with_caliper,type="jitter")

**Figure 5:**
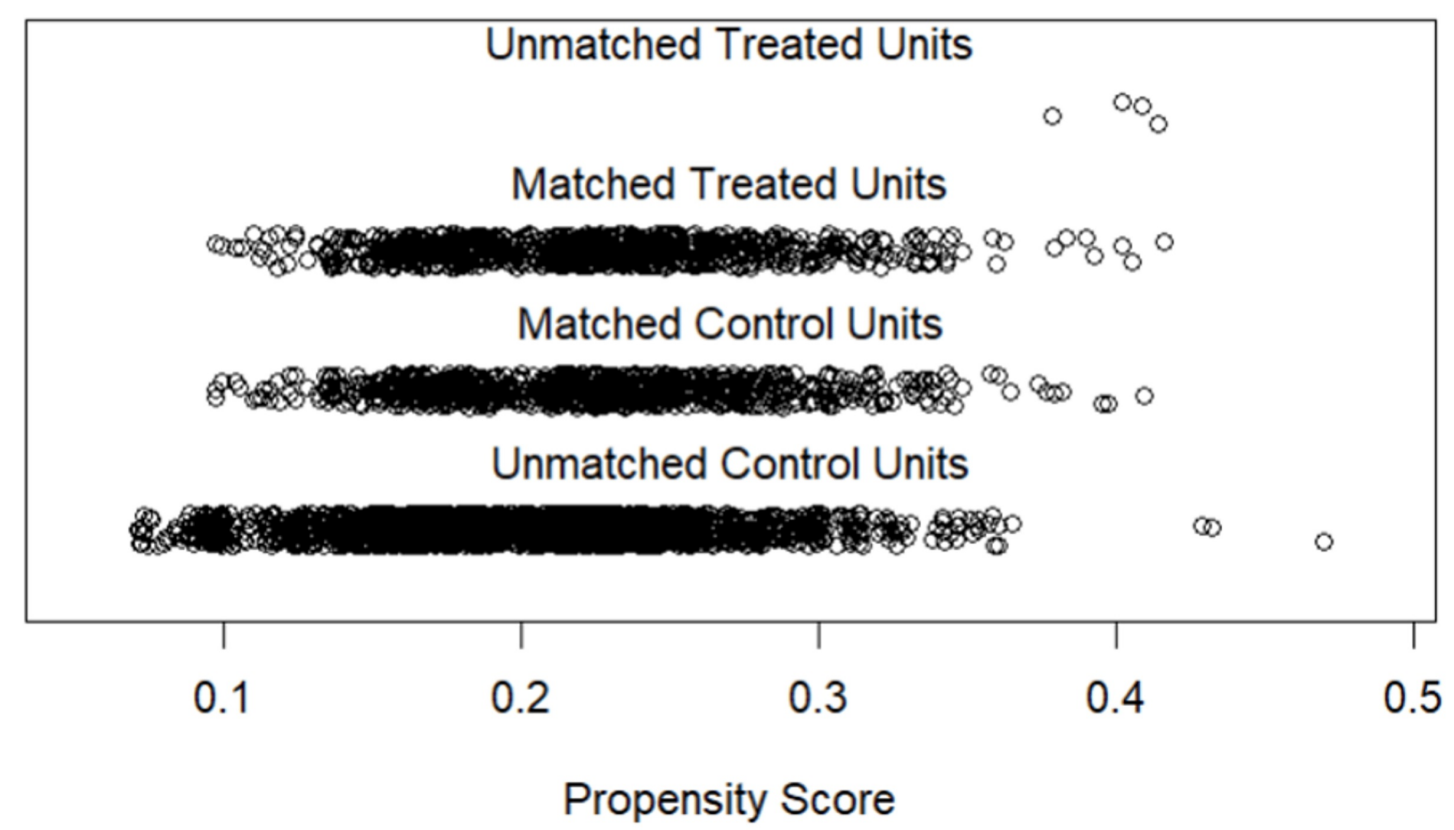
Visualization of distribution of propensity scores using jitter plots (Caliper = 0.2).

In Figure 5 below, we can see that we have four observations under unmatched treated units who did not have a match. These observations were unmatched because we set a caliper, and no suitable matches were found within the allowable propensity score range for these individuals. Based on the summary table (Table 5), we can now see that the sample size has been reduced to 853 from 857 after we set the caliper to 0.2, meaning that the maximum allowed difference between propensity scores is 0.2. In our set of observations, because four treated observations did not find a match within that caliper, it was excluded after the matching.

## Discussion

The intent of this article is to serve as an introductory step-by-step tutorial on how to conduct PSM for an observational study and highlight the use of RStudio as an example using the 2022 NHIS dataset. This is a pedagogical working example and is not intended to be used as a guide for using nationally representative analysis, as datasets like NHIS require incorporation of the survey weights, cluster, and strata, which is beyond the scope of this paper. It is also important to note that weighting is used in national datasets to make the results more representative of the target population. Using survey weights helps minimize bias and improve the validity of survey results. The results obtained from this tutorial should be interpreted as unweighted estimates and are appropriate only for demonstrating the steps and procedures of propensity score estimation.

We have demonstrated each stage of the PSM process, from data handling to estimating propensity scores, as well as the matching process. We have provided readers with guidance on creating a matched dataset using 1:1 nearest neighbor matching with a caliper of 0.2, and we also demonstrated how matching helps balance the dataset using SMDs with a threshold of 0.1. We have used a tabular method to show the difference in the SMD before and after matching. Future studies could incorporate graphical balance diagnostics, such as a *Love plot*, to complement the tabular SMD comparisons presented here.

One of the major limitations of this tutorial is the use of CCA, where we removed all the missing values of variables, which could lead to bias if the data are not missing completely at random (MCAR) [15]. Alternative approaches to CCA in PSM can be the missing indicator method, multiple imputation, and combining multiple imputation and the missing indicator method. Researchers should also examine patterns of missingness and decide which method is appropriate for the dataset they intend to use.

We have demonstrated that PSM can improve balance in measured covariates and may help reduce bias from observed confounders. It is important to note that PSM cannot control unmeasured variables. Additionally, results can be sensitive to covariate selection and may yield different matched samples and conclusions. Finally, the utilization of a caliper needs to be approached very carefully. While calipers can improve the quality of matches, they can also reduce the number of matched units, reducing the sample size and power of the study.

An important consideration when using national datasets like the NHIS dataset is its complex survey design, which includes stratification, clustering, and sampling weights intended to give nationally representative estimates of the US population. Ignoring the sampling weights in NHIS can limit the generalizability of the findings. This tutorial does not incorporate survey weights in the propensity score model or matching procedure. This example is only for pedagogical purposes. Furthermore, appropriate assessment of common support and overlap in propensity scores is essential to ensure that treated and control groups are comparable. This can be evaluated using graphs such as propensity score distributions before and after matching.

Previous articles have been published to provide readers with guidance on incorporating propensity scores into their analyses, and we highly recommend reading these resources for those seeking an in-depth understanding of PSM [15,17,18]. For example, Randolph and colleagues provided a tutorial on conducting PSM in R with the *MatchIt* package; however, their work was rooted in the assessment of educational programs [19]. It is also equally important to acknowledge the ongoing methodological debate surrounding PSM. King and Nielsen demonstrated that PSM could increase imbalance, model dependence, and bias [20].

Zhao and colleagues were able to provide a thorough guide for users interested in incorporating propensity scores in newer techniques grounded in machine learning, yet their examples do not incorporate freely accessible data [15]. Likewise, Lee et al. provided a practical PSM tutorial using R and Rex, an Excel add-in interface for users less familiar with programming [18]. Their article demonstrates variable selection, propensity score estimation, matching, balance assessment, and treatment effect evaluation, while also discussing advanced estimation options such as regularized regression and machine learning models. In contrast, our tutorial is more introductory and applies PSM in RStudio using the publicly available 2022 NHIS dataset. The previous works by Lee and Zhao, while comprehensive, are likely more suitable for experienced researchers and programmers who are looking to sharpen skills, which are already advanced, and more than those who are beginning to learn more about R and PSM [15,18]. In addition, the present tutorial utilizes publicly available data from the NHIS, which facilitates replication by any user with access to the internet. By using an example from a national survey and grounding the tutorial in a health services research question, this tutorial is directly applicable to epidemiologists and health services researchers who commonly work with public datasets.

## Conclusions

The applied example was chosen for its public health relevance and the availability of the dataset, with the goal that researchers working with the NHIS or similar nationally representative surveys will find the steps useful as a starting point for their own projects. All codes used in this tutorial are reproducible using freely available software and publicly downloadable data, with no specialized computing resources required. While the steps demonstrated in this tutorial may be useful for guiding similar studies, they should be tailored carefully based on the data characteristics, weighting strategy, and study design of other projects.

Researchers should be aware that PSM is not universally the optimal confounding control strategy. The goal of this tutorial is not to advocate for PSM but to assist researchers with the knowledge to implement it correctly and evaluate its outputs critically. The main intention of this paper is to create a simplified tutorial and explanation for health researchers to lower the barrier to entry for those new to RStudio as well as matching methods.

## Appendices

**Table 6:** Abbreviations.

Abbreviation	Full form
PSM	Propensity Score Matching
PS	Propensity Score
NHIS	National Health Interview Survey
SMD	Standardized Mean Difference
RCT	Randomized Controlled Trials
IPTW	Inverse Probability of Treatment Weighting
CSV	Comma-Separated Values
URBRRL	Urban-Rural Classification Variable in NHIS
VIRAPP12M_A	Telehealth Use in Past 12 Months (NHIS variable)
AGEP_A	Age (NHIS variable)
SEX_A	Sex (NHIS variable)
RACEALLP_A	Race (NHIS variable)
EDUCP_A	Education Level (NHIS variable)
MARITAL_A	Marital Status (NHIS variable)
HICOV_A	Health Insurance Coverage (NHIS variable)
COMDIFF_A	Communication Difficulty (NHIS variable)
POVRATTC_A	Income-to-Poverty Ratio (NHIS variable)
HISP_A	Hispanic Ethnicity (NHIS variable)
